# The Complete Chloroplast Genome Sequence of Date Palm (*Phoenix dactylifera L.*)

**DOI:** 10.1371/journal.pone.0012762

**Published:** 2010-09-15

**Authors:** Meng Yang, Xiaowei Zhang, Guiming Liu, Yuxin Yin, Kaifu Chen, Quanzheng Yun, Duojun Zhao, Ibrahim S. Al-Mssallem, Jun Yu

**Affiliations:** 1 The Date Palm Genome Project (DPGP), King Abdulaziz City for Science and Technology (KACST), Riyadh, Kingdom of Saudi Arabia; 2 Key Laboratory of Genome Sciences and Information, Beijing Institute of Genomics, Chinese Academy of Sciences, Chaoyang District, Beijing, China; 3 Department of Biotechnology, College of Agriculture and Food Sciences, King Faisal University, Al-Hssa, Hofuf, Kingdom of Saudi Arabia; The J. Craig Venter Institute, United States of America

## Abstract

**Background:**

Date palm (*Phoenix dactylifera L.*), a member of Arecaceae family, is one of the three major economically important woody palms—the two other palms being oil palm and coconut tree—and its fruit is a staple food among Middle East and North African nations, as well as many other tropical and subtropical regions. Here we report a complete sequence of the data palm chloroplast (cp) genome based on pyrosequencing.

**Methodology/Principal Findings:**

After extracting 369,022 cp sequencing reads from our whole-genome-shotgun data, we put together an assembly and validated it with intensive PCR-based verification, coupled with PCR product sequencing. The date palm cp genome is 158,462 bp in length and has a typical quadripartite structure of the large (LSC, 86,198 bp) and small single-copy (SSC, 17,712 bp) regions separated by a pair of inverted repeats (IRs, 27,276 bp). Similar to what has been found among most angiosperms, the date palm cp genome harbors 112 unique genes and 19 duplicated fragments in the IR regions. The junctions between LSC/IRs and SSC/IRs show different features of sequence expansion in evolution. We identified 78 SNPs as major intravarietal polymorphisms within the population of a specific cp genome, most of which were located in genes with vital functions. Based on RNA-sequencing data, we also found 18 polycistronic transcription units and three highly expression-biased genes—*atpF*, *trnA-UGC*, and *rrn23.*

**Conclusions:**

Unlike most monocots, date palm has a typical cp genome similar to that of tobacco—with little rearrangement and gene loss or gain. High-throughput sequencing technology facilitates the identification of intravarietal variations in cp genomes among different cultivars. Moreover, transcriptomic analysis of cp genes provides clues for uncovering regulatory mechanisms of transcription and translation in chloroplasts.

## Introduction

The chloroplast (cp), believed to arise from endosymbiosis between a photosynthetic bacterium and a non-photosynthetic host [1], is the photosynthetic organelle that provides essential energy for plants and algae. A given cell, such as that of a plant leaf, often contains 400 to 1,600 copies of cp genome [2]. Other biological activities also take place in chloroplast including the production of starch, certain amino acids and lipids, vitamins, certain pigments in flowers and several key pathways of sulfur and nitrogen metabolism [3]. In angiosperm, most cp genomes are circular DNA molecules ranging from 120 to 160 kb in length and have a quadripartite organization consisting of two copies of inverted repeats (IRs) of ∼20–28 kb in size, which divides the rest of genome into a large-single-copy region (LSC; 80–90 kb) and a small-single-copy (SSC; 16–27 kb) region. Usually, the gene content of angiosperm cp genome is rather conserved, encoding 4 rRNAs, ∼30 tRNAs, and ∼80 unique proteins [4]. Since the completion of the first cp genome of tobacco (*Nicotiana tabacum*) [5], there have been many discoveries on rearrangements and IR expansions within cp genomes, perhaps the most remarkable one is that of *Pelargonium x hortorum*, in which numerous rearrangements and large IR expansions were found [4]. Gene losses have been found frequently in angiosperm cp genomes [6], [7], [8].

The date palm (*Phoenix dactylifera, L*.), an Arecaceae family member, is one of the most economically important woody plant cultivated in Middle East and North Africa [9]. Its fruit provides staple food across Asian (mainly in Arabian Peninsula, Iran, and Pakistan), African, Europe, and American tropics. A recent report suggests that there are more than 340 cultivars in Saudi Arabia and nearly 2,000 cultivars around the world [10]. The total number of date palm trees grown in the world is ∼100 million, producing 15 million metric ton fruit each year [11]. The cultivated hybrids of date palm are mostly diploid (2n = 36) and propagated from offshoots [12]. Because cp genome is maternally inherited [13], deeper knowledge about its structure, sequence variation, and evolution provides useful information for developing propagation technologies, such as cytoplasmic breeding and transgenic insertion.

In the past 10 years, we have witnessed a dramatic increase in the number of complete cp genomes. Up to now, 132 complete land plant cp genomes have been deposited in GenBank Organelle Genome Resources, albeit only 22 of them are monocot cp genomes and most of these genomes were sequenced using capillary sequencers [14]. With the emergence of next-generation sequencers, new approaches for genome sequencing have been gradually proposed due to their high-throughput, time-saving, and low-cost advantages [15], [16], [17]. Here, we report the complete cp genome sequence of date palm, the first member of the Arecaceae family from an elite cultivar *Khalas* (Al-Hssa Oasis, Saudi Arabia), using one of the next-generation sequencing method – pyrosequencing (Roche GS FLX). We also describe details in genome assembly, annotation, and comparative analysis, as well as information on sequence variations and trancriptomics for the date palm cp genome.

## Results and Discussion

### 1. Genome assembly and validation

Using Roche GS FLX system, we carried out five sequencing runs to generate 4,169,506 raw reads (347 bp in average read length) for the project. After screening the reads through alignment with reference cp genomes and extensive contig extension efforts (see Materials and Methods for details), we collected 369,022 cp-genome-related reads (8.8% of total reads and an average of 384 bp in length), reaching 1,081x coverage on average over the cp genome. After validating the homopolymer regions and the junctions between single-copy and IR regions with PCR-based confirmation, we obtained a complete cp genome sequence of 158,462 bp in length.

Typically, two types of errors are characteristic in pyrosequencing data processing; one is associated with contig ends and the other involves heterogeneous insertion/deletions (Indels) arisen from homopolymeric repeats [17]. The first is basically a sequence quality issue and usually overcome by increasing coverage (here we have 369,022 cp reads) and removing low-quality reads. The second is intrinsic to pyrosequencing and can not be improved by increasing coverage; we therefore performed alternative types of experiments to correct the erroneous homopolymer calls found in the assembly. Based on the homopolymer (n> = 7) distribution in the preliminary cp genome assembly, we designed 151 pairs of PCR primers to validate all homopolymer runs in the entire assembly by using capillary sequencing (Table S1). The result was very satisfactory where we added 117 base pairs in 108 homopolymers, and all previously uncertain or missed nucleotides, either A or T (except for one homopolymer run that has 13 cytosines) were satisfactorily validated. These recovered nucleotides appeared larger in number as compared to what encountered when sequencing *Mungbean* cp genome, where only 49-bp sequences were lost in the initial assembly (74.6x raw data) [15]. Since we have 161 homopolymers above 7 bp, and 181 equal to 7 bp, the potential errors are too large to not handle them with care and a simple increase of sequence coverage is not likely to be useful [17].

### 2. Genome features

**Figure 1:**
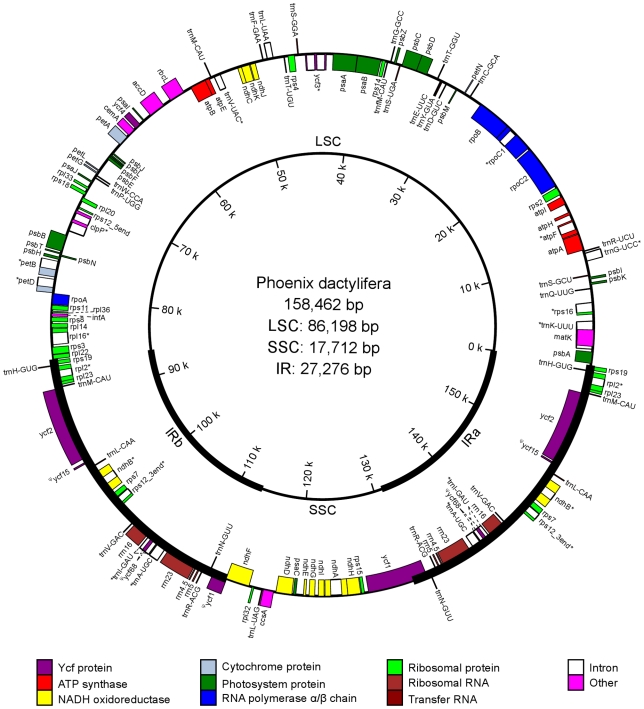
The map of date palm cp genome sequence. Genes shown outside the outer circle are transcribed clockwise, whereas those shown inside are transcribed counterclockwise. The thick line indicates IRs. The genome coordinate is shown in the inner circle. Genes belonging to different groups are color-coded (Ψ, pseudogene).

The date palm cp genome is a typical circular double-stranded DNA molecule, and it shares a common quadripartite structure with the vast majority of other angiosperms: a pair of IRs (27,276 bp) separated by the LSC (86,198 bp) and SSC regions (17,712 bp) (Figure 1). It encodes 131 predicted functional genes; 112 are unique and 19 are duplicated in the IR regions. Among the 112 unique genes, we identified 79, 29, and 4 protein-coding, transfer RNA, and ribosomal RNA genes, respectively. 50.93%, 1.79%, and 5.71% of the genome sequence encode proteins, tRNAs, and rRNAs, respectively, whereas the remaining 41.57% are non-coding and filled with introns, intergenic spacers, and pseudogenes. Similar to other cp genomes [18], [19], the date palm cp genome is also AT-rich (62.77%), and the values vary slightly among defined sequences of non-coding, protein-coding, tRNA, and rRNA, where their A+T contents are 66.60%, 61.03%, 57.94%, and 52.19%, respectively.

Similar to tobacco cp genome, the date palm cp genome has 18 intron-containing genes among the 112 unique genes; almost all are single-intron except for two genes, *ycf3*, and *clpP*, whose exons are separated by two introns. The gene *rps12* is trans-spliced; one of its exons is in the LSC region (5′) and the two reside in the IR regions separated by an intron. The introns of all protein-coding genes share the same splicing mechanism as Group II introns [20]. In the IR regions, both *ycf15* and *ycf68* became pseudogenes duo to internal stop codons identified in their coding sequences (CDS). Specifically, the CDS of *ycf15* is interrupted by a stop codon at downstream (57 bp away from the start codon), whereas several stop codons appeared in *ycf68* gene. Similar mutations have also been known to happen in the cp genomes of other species [19]. Another pseudogene is *ycf1* in the boundary of IRb and SSC because of the incomplete duplication of the normal copy of *ycf1* at the IRa and SSC boundary (Figure 1).

A total of 22,950 codons represent the coding capacity of all protein-coding genes of date palm cp genome (Table 1). Among these codons, 2001 (8.72%) encode for isoleucine and 271 (1.18%) for cysteine, which were the most and the least amino acids, respectively. These extremes are the same as what in nuclear genomes. The base compositions at each codon position are slightly biased: 54.4%, 61.8%, and 70.6% for the first to the third, respectively.

**Table 1:** Codon usage and codon-anticodon recognition pattern for tRNA in date palm chloroplast genome.

Amino acid	Codon	NO.	RSCU	tRNA	Amino acid	Codon	NO.	RSCU	tRNA
Phe	UUU	836	1.29		Ser	UCU	498	1.69	
	UUC	462	0.71	trnF-GAA		UCC	289	0.98	trnS-GGA
Leu	UUA	734	1.89	trnL-UAA		UCA	371	1.26	trnS-UGA
	UUG	500	1.29	trnL-CAA		UCG	162	0.55	
Tyr	UAU	700	1.6		Cys	UGU	207	1.53	trnC-GCA
	UAC	177	0.4	trnY-GUA		UGC	64	0.47	
Ter	UAA	37	0		Ter	UGA	20	0	
Ter	UAG	22	0		Trp	UGG	392	1	trnW-CCA
Leu	CUU	473	1.22		Pro	CCU	367	1.57	
	CUC	162	0.42			CCC	185	0.79	
	CUA	314	0.81	trnL-UAG		CCA	275	1.18	trnP-UGG
	CUG	150	0.39			CCG	107	0.46	
His	CAU	423	1.54		Arg	CGU	322	1.39	trnR-ACG
	CAC	127	0.46	trnH-GUG		CGC	78	0.34	
Gln	CAA	608	1.5	trnQ-UUG		CGA	307	1.32	
	CAG	200	0.5			CGG	106	0.46	
Ile	AUU	959	1.44		Thr	ACU	465	1.56	
	AUC	415	0.62	trnI-GAU		ACC	223	0.75	trnT-GGU
	AUA	627	0.94			ACA	382	1.28	trnT-UGU
Met	AUG	545	1	trn(f)M-CAU		ACG	125	0.42	
Asn	AAU	857	1.59		Ser	AGU	361	1.23	
	AAC	223	0.41	trnN-GUU		AGC	84	0.29	trnS-GCU
Lys	AAA	884	1.5	trnK-UUU	Arg	AGA	445	1.92	trnR-UCU
	AAG	294	0.5			AGG	135	0.58	
Val	GUU	470	1.44		Ala	GCU	559	1.83	
	GUC	162	0.5	trnV-GAC		GCC	188	0.62	
	GUA	496	1.52	trnV-UAC		GCA	347	1.14	trnA-UGC
	GUG	181	0.55			GCG	127	0.42	
Asp	GAU	741	1.6		Gly	GGU	550	1.4	
	GAC	184	0.4	trnD-GUC		GGC	137	0.35	trnG-GCC
Glu	GAA	935	1.53	trnE-UUC		GGA	633	1.61	trnG-UCC
	GAG	290	0.47			GGG	251	0.64	

**Figure 2:**
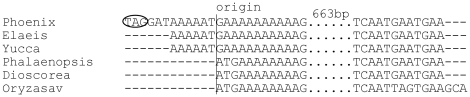
A multiple alignment of *cemA* genes from representative monocot cp genomes. The start codon is assigned as origin. The possible stop codons in the upstream regions are marked by ellipse. 663-bp less-relevant aligned sequences were omitted.

There are some exceptional cases in start codons. We identified two ACG as start codons in *rpl2* and *ndhD* and one GUG start codon in *rps19*. The non-canonical starts have been detected in other angiosperms [19] and even in fern-like plant *Alsophila spinulosa*, where 20 genes start with ACG [18]. We also found an unconventional start codon in *cemA* that encodes a heme-binding protein functioning in the inner chloroplast envelope membrane [21]. Multiple alignments of cp genomes show that there is an 8-bp homopolymer (AAAAAAAA) in the downstream of its start codon sequences commonly found among certain monocots, such as rice. However, in date palm (also in oil palm and *Yucca*), this homopolymer is 10 bp in length, resulting a 2-bp coding frame shift that changes ATG to AAT and GAA (Figure 2). Moreover, a stop codon—TAG—12 bp in the upstream sequence makes the start codon of *cemA* in date palm rather ambiguous. Our transcriptome analysis demonstrated that its mRNA is in a polycistronic transcription unit, together with *psaI*, *ycf4*, and *petA* (Part 7). However, whether this gene is translatable to protein or not remains unclear due to the obscurity of its start codon. To make further inspection to this gene, we aligned its orthologs from 74 angiosperms and 3 gymnosperms (*Ginkgo*, *Pinus* and *Welwitschia*) and found a start codon among all other species either at the origin or the upstream other than what in date palm, oil palm, *Yucca* and *Lemna* (Figure S1). Although ATG is typical the start codon, GUG is also used in 14 species. Most of the 14 species are either basal angiosperms or gymnosperms except two in Acoraceae family (*Acorus calamus* and *Acorus americanus*) [22]. These results suggest that the start codon of *cemA* gene must have endured a strong selective pressure during the early evolution of angiosperms but it is somewhat relaxed among certain plant taxa.

### 3. Gene order and content

Since the tobacco cp genome is often regarded to be unarranged [5], we compared our assembly with it and observed a high degree of synteny between the two cp genomes. Two minor exceptions were found—additional copies of *rps19* and *trnH-GUG* around the junction between LSC and IRs— due to IR expansions in the date palm cp genome. The gene content is also nearly identical with that of tobacco; only a single gene, namely *infA* common among monocots, appeared degenerated into a pseudogene in tobacco. However, structural rearrangements and gene loss-and-gain events are quite often among other monocot cp genomes, mainly found in Poaceae family, where the canonical order is disrupted by three inversions in their LSC regions and *rpl23* are translocated from IR to LSC regions [23]. Indels are also frequently found in Poaceae such as intron-loss in *rpoC1* and insertion in *rpoC2* [24]. Other variations are gene-loss (deletion or becoming pseudogene) of *accD*, *ycf1*, and *ycf2* in Poaceae cp genomes [25]. We calculated the vestigial lengths of these three pseudogenes and found that their sequence variations can explain in part the cp genome size differences between Poaceae species (137,851 bp in average) and date palm, since the average size of the Poaceae cp genomes is ∼14 kb shorter than that of date palm. In addition, gene-loss event often occurs in other monocot families (cp genomes of 22 monocot species are available in public databases and 15 of them are Poaceae species). There is only one Typhaceae cp genome published recently, which has the same gene content and order as date palm [26]. Among the remaining cases, *Lemna* and *Dioscorea*, each lost a single gene: *infA* and *rps16*, respectively; two Acoraceae members lost *accD* [6], [7], [24] and *Phalaenopsis* and *Oncidium* lost most of *ndh* genes [8], [27]. Rearrangements also occurred in *Dioscorea*, such as the inversion of SSC. Nevertheless, similar to that of tobacco, date palm cp genome appeared less rearranged and having very limited gene loss-and-gain especially when compared to these monocots.

### 4. IR expansion and evolutionary implications

Around the borders of JLB and JLA, date palm has the same structure as what in Poaceae cpDNAs; specifically, in JLB, *rpl22* and its 5′-end adjacent *rps19* are completely fell in LSC and IRb, respectively, whereas in JLA, another copy of *rps19* in IRa adjoins its 3′-end to *psbA* in LSC. However, surrounding JSB and JSA, the gene order of the date palm assembly is similar to those of *Amborella* and certain dicots (i.e., tobacco, *Panax*, and *Arabidopsis*), namely JSB locates between *ycf1* pseudogene and *ndhF*, whereas JSA resides in the 3′ region of the normal *ycf1* gene.

**Figure 3:**
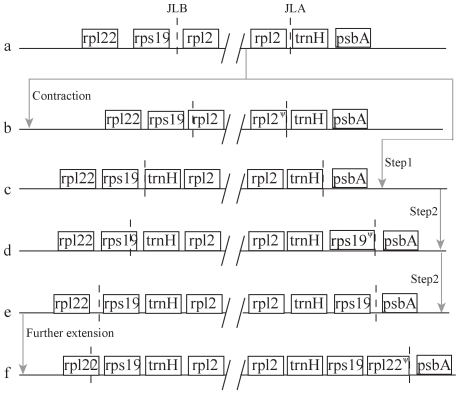
Expansion of IR into the LSC region. The dashed lines depict the junction boundaries. The arrowed grey lines indicate the process of IR expansions. a, *Amborella* and *Nicotiana*; b, *Lemna*; c, intermediate; d, *Acorus* and *Dioscorea*; e, date palm, *typha,* and all Poaceaes; f, *Phalaenopsis* and *Oncidium*.

Among other monocots, various degrees of IR to LSC expansions were identified. In *Dioscorea* and *Acorus*, only the 5′-end of *rps19* are included in IRb to generate a *rps19* pseudogene in IRa [28], whereas in *Lemna*, a contraction was detected as IRb shrunk into the 3′-end of *rpl2* [6]. Other than *Lemna*, all other monocots studied thus far possess a copy of *trnH-GUG* (in 5′-end of *rps19* in IRb) that is absent in tobacco and *Amborella*. Therefore, based on the two-step hypothesis of IR expansion [6], we deduced that the formation of IR-LSC boundaries among monocots (except *Lemna*) occurs in the following manner: *trnH-GUG* is duplicated in IRb as the first step of inclusion of *trnH-GUG* to IRa; and along with the second expansion step in recruiting rps19 to IRb, another copy of *rps19* (or pseudogene) is generated in IRa. However, in *Phalaenopsis* and *Oncidium*, the expansion was unusual where IRb extended to the 3′-end of *rpl22* gene [8], [27] (Figure 3).

**Figure 4:**
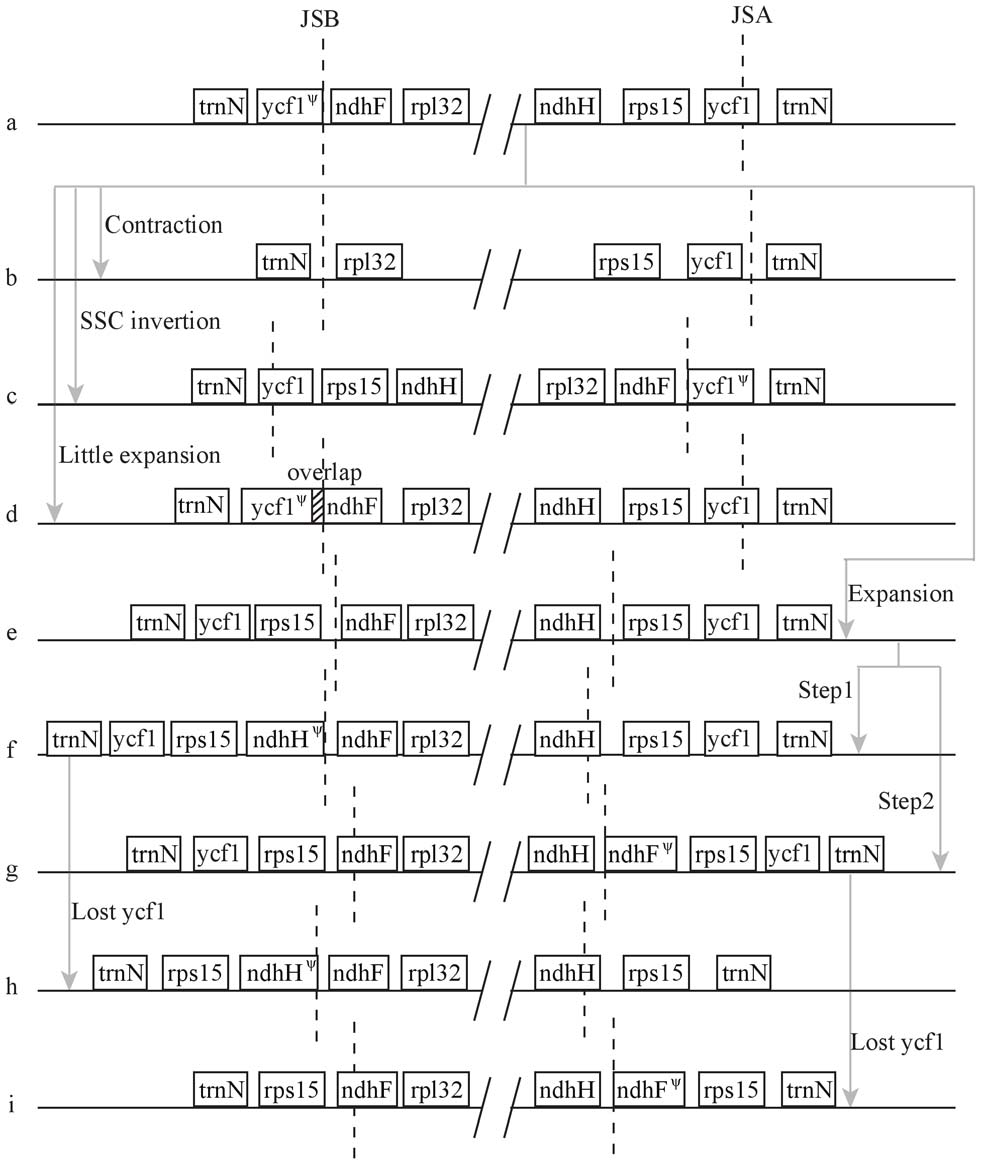
Expansion of IR into the SSC region. The dashed lines depict the junction boundaries. The arrowed grey lines indicate the process of IR expansions. a, *Amborella*, *Nicotiana*, *typha and* two Acoraceae members; b, *Phalaenopsis*; c, *Dioscorea*; d, date palm and *Arabidopsis*; e, intermediate; f, *Lemna*; g, intermediate; h, most Poaceaes; i, Panicoideae subfamily including *Coix*, *Maize, Saccharum and Sorghum*.

At the two boundaries of SSC, the general structure revealed in *Amborella* and dicots (i.e., tobacco, *Panax* and *Arabidopsis*) is that *ycf1* spans JSA and *ycf1* pseudogene adjacent to JSB. In monocots, two Acoraceae members share this structure; and similar to *Arabidopsis*, a small expansion occurred in date palm, which formed an overlap between *ndhF* and *ycf1* pseudogene (55 bp in date palm) in JSB in date palm [28]. In *Dioscorea*, this structure is inverted—the normal *ycf1* in JSB and the corresponding *ycf1* pseudogene in JSA—because of the inversion of SSC. In *Phalaenopsis*, there is a short contraction of IRs where *ycf1* is completely included in SSC [8]. A special case in monocots is found in *Lemna*, its expansion pattern of IRs to SSC is similar to that in Poaceae family if we exclude the complete duplicate of *ycf1* [6]. Since we can find the symmetrically degenerated *ycf1* in several Poaceae cp genomes (i.e., the position 99,623–100,458 and 114,660–115,495 in IRb and IRa, respectively, in *Oryza sativa*) in the corresponding position of *Lemna*, we speculate that in the beginning, the ancestors of Poaceae experienced similar IR expansion into SSC. Therefore, there are two types in IR/SSC junctions in the early evolution of monocots: one is little changed such as the case of date palm and Acoraceae members, which has no obvious expansion to share similar structure with *Amborella* or tobacco; the other experienced apparent expansion to firstly include the whole duplicates of *rps15* and *ycf1* in IRb, and thereafter, two possible expansions occur. The first one is the incorporation of the 5′-end of *ndhH,* resulting in an incomplete copy of *ndhH* pseudogene in IRb. This expansion happened in *Lemna* as well as probably among most Poaceaes. The second type is found in *Panicoideae* subfamily of Poaceae cp genomes and IRb expand into SSC to include the 3′-end of *ndhF*, accompanying with an incomplete copy of *ndhF* pseudogene in JSA. After these expansions, *ycf1* became nonfunctional and resulted in the present Poaceaes structure (Figure 4).

Gene order in the four junctions has been known to vary among different cp genomes due to the expansion or contraction of the IR regions [8], [19], [28], [29]. The general process of IR changes has also been surveyed for monocots cp genomes [6], [26]. We proposed a most likely evolution rout of IRs and summarized patterns of expansions between junctions of IR/LSC and IR/SSC in comparison to *Amborella* and *tobacco*. The basal monocots in our analysis are members of Acoraceae family, whereas the higher monocots belong to Poaceae. However, in the IR/LSC junctions, *Lemna* has a more contracted IR structure than *Acrous* and the basal angiosperm— *Amborella* [6], [22]; and larger expansion occurred in Orchidaceae than in Poaceae. In junctions of IRs/SSC, we observed obvious expansion in *Lemna* and all Poaceae members, whereas little expansion was noticed in all other monocots, and there is even an IR contraction in *Phalaenopsis*. Therefore, the expansion rate may not be in accordance with the taxonomic relationship among monocots.

### 5. Repeat structure and small inversions

Using REPuter, we identified 11 forward and inverted repeats 30 bp or longer with a sequence identity greater than 90% (Table 2). Three pairs of forward repeats were found in the coding region, while seven pairs were identified either in intergenic or intronic regions. The remaining was mostly in the two *trnS* genes. Compared to other species, the number of repeats in date palm cp genome is fairly low; for example, in Poaceae families, there are 19–37 forward and inverted repeats with the size ranging from 30 bp to 60 bp [30]; and in some dicots, such as *Gossypium* [31] and *Citrus* [3], the number of repeats are 54 and 29, respectively. The lengths of repeats in the date palm assembly are also much shorter, and the longest repeat is only 39 bp in length, whereas a much longer 91-bp repeat was found in Poaceae family [30].

**Table 2:** Repeated sequences in the date palm chloroplast genome.

Repeat number	Repeat size(bp)	Repeat1 start	Repeat2 start	Repeat type	Repeat1 location	Repeat2 location
1	35	8334	45650	I	ItrnS-GCU	ItrnS-GGA
2	39	44165	143194	I	intron (ycf3)	IGS(trnV-GAC - rps12_3end)
3	31	84427	112085	I	intron (rpl16)	IGS(trnN-GUU - ycf1)
4	30	126644	126677	I	IGS (rps15 - ycf1)	IGS (rps15 - ycf1)
5	30	8342	35995	F	trnS-GCU	trnS-UGA
6	34	39250	41474	F	psaB	psaA
7	39	44166	101428	F	intron (ycf3)	IGS (rps12_3end - trnV-GAC)
8	30	69830	69863	F	IGS (rpl33 - rps18)	IGS (rpl33 - rps18)
9	30	83886	120587	F	intron (rpl16)	IGS (psaC - ndhE)
10	31	84428	132545	F	intron (rpl16)	IGS (ycf1 - trnN-GUU)
11	38	93972	94020	F	ycf2	ycf2

I partly in the IGS region; I-Inverted, F-forward, IGS-Intergenic spacer region.

**Figure 5:**
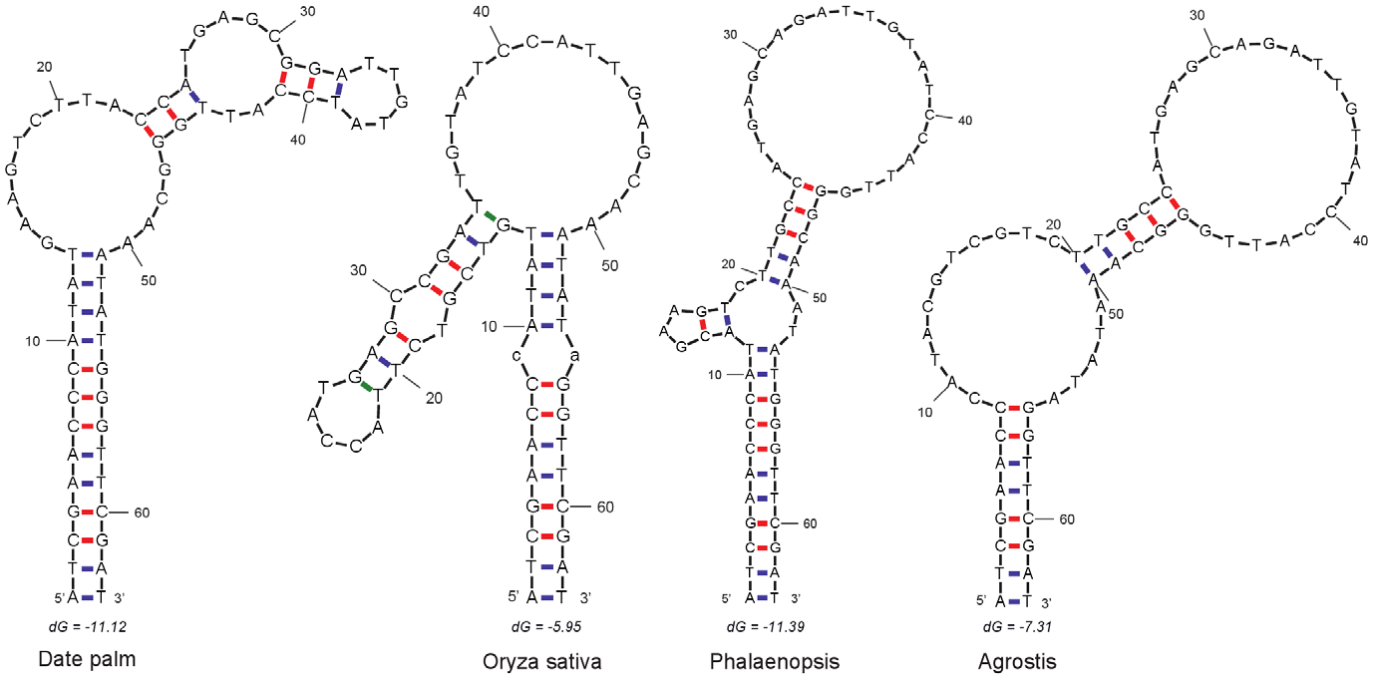
Folded stem-loop structures of the new SI from four representative species. Free energy values (dG) are shown for SI in each species.

Small inversions or SIs between IRs are quite interesting upon examination. SIs vary in length from 5 to 50 bp, and are flanked by a pair of IRs ranging from 11 to 24 bp in size. SIs can generally be determined through pair-wise comparison between the sequences from closely related taxa. A comprehensive result of 16 SIs has been reported previously for plant chloroplasts [32]. Additional two SIs have been found recently in the intergenic region of *psbA-*t*rnH* and *psbC*-*trnS* [33], [34]. Here, we also identified a new SI in date palm cp genome after performing alignments with its orthologs in other monocots (Table 3). This SI is located in *psaB* coding region, 63 bp in length, and has the stem and loop being 13 and 37 bp, respectively, in most analyzed cases. The loop regions in the aligned sequences have the same orientation across different taxa and have at least 90% sequence identity to that of data palm. Sequence variations in IR region mostly occurred as a G-to-A single nucleotide polymorphism (SNP), which decrease the stability of the secondary structures. In *Lemna* and *Acrous*, there were three SNPs found in the stem region, making the stem-loop structure rather unstable as their free energy values are −1.49 and −1.1, respectively; in tobacco and *Panax*, numerous variations disrupt the secondary structure seriously. Different loop configurations of the SIs are generated when folding to secondary structure due to their large size and variations in the loop sequences. The alteration is conspicuous in *Phalaenopsis* and *Agrostis*, whose loops are even longer (41 and 47 bp, respectively) than most of others (37 bp) due to the variation near the two ends of the loop in the stem region (Figure 5). However, this did not affect the free energy of their secondary structures. *Phalaenopsis* has almost identical free energy (−11.39) with date palm (−11.12), whereas the free energy of *Agrostis* (−7.31) is a little lower than that of the *Pooideae* subfamily (−9.14).

**Table 3:** The location and sequences of small inversion in *psaB*.

Taxa	Genome coordination	aSequence alignment	Free energy (ΔG)
Phoenix	38,667–38,729	ATCGAACCCATAT**GAAGTCTTACCATGAGCGGATTGTATCCATTGGGCAA**ATATGGGTTCGAT	−11.12
bPhalaenopsis	39,524–39,586	ATCGAACCCAT**ACGAAGTCTTGCCATGAGCAGATTGTATCCATTGGGCAAAT**ATGGGTTCGAT	−11.39
bOncidium	37,640–37,702	ATCGAACCCAT**ACGAAGTCTTGCCATGAGCAGATTGTATCCATTGGGCAAAT**ATGGGTTCGAT	−11.39
Typha	40,779–40,839	ATCGAACCCATAT**GAAGTTTTACCATGAGCAGATTGTATCCATTGGGCAA**ATATGGGTTCGAT	−10.05
Brachypodium	37,341–37,403	ATCGAACCCATAT**GTCGTCTTGCCATGAGCAGATTGTATCCATTGGGCAA**ATAT**a**GGTTCGAT	−9.14
Festuca	37,657–37,719	ATCGAACCCATAT**GTCGTCTTGCCATGAGCAGATTGTATCCATTGGGCAA**ATAT**a**GGTTCGAT	−9.14
Hordeum	38,178–38,240	ATCGAACCCATAT**GTCGTCTTGCCATGAGCAGATTGTATCCATTGGGCAA**ATAT**a**GGTTCGAT	−9.14
Lolium	37,293–37,355	ATCGAACCCATAT**GTCGTCTTGCCATGAGCAGATTGTATCCATTGGGCAA**ATAT**a**GGTTCGAT	−9.14
Triticum	38,014–38,076	ATCGAACCCATAT**GTCGTCTTGCCATGAGCAGATTGTATCCATTGGGCAA**ATAT**a**GGTTCGAT	−9.14
bAgrostis	37,863–37,925	ATCGAACC**CATACGTCGTCTTGCCATGAGCAGATTGTATCCATTGGGCAAATATA**GGTTCGAT	−7.31
Dioscorea	37,740–37,802	ATCGAACCCATAT**GAAGTCTTACCATGAGCAGATTGTATCCATTGGGCAA**ATATGGGTTC**a**AT	−7.06
Anomochloa	39,677–39,739	ATCGAACC**c**ATAT**GTCGTCTTACCATGAGCGGATTGTATCCATTGGGCAA**ATAT**a**GGTTCGAT	−6.12
Oryza.niv	37,482–37,544	ATCGAACCCATAT**GTCGTCTTACCATGAGCCGATTGTATCCATTGAGCAA**ATAT**a**GGTTCGAT	−5.95
Oryza.sat	37,548–37,610	ATCGAACCCATAT**GTCGTCTTACCATGAGCCGATTGTATCCATTGAGCAA**ATAT**a**GGTTCGAT	−5.95
Bambusa	39,617–39,679	ATCGAACCCATAT**GTCGTCTTACCATGAGCAGATTGTATCCATTGGGCAA**ATAT**a**GGTTCGAT	−5.2
Coix	39,617–39,679	ATCGAACCCATAT**GTCGTCTTACCATGAGCAGATTGTATCCATTGGGCAA**ATAT**a**GGTTCGAT	−5.2
Dendrocalamus	39,782–39,844	ATCGAACCCATAT**GTCGTCTTACCATGAGCAGATTGTATCCATTGGGCAA**ATAT**a**GGTTCGAT	−5.2
Saccharum	40,453–40,515	ATCGAACCCATAT**GTCGTCTTACCATGAGCAGATTGTATCCATTGGGCAA**ATAT**a**GGTTCGAT	−5.2
Sorghum	40,672–40,734	ATCGAACCCATAT**GTCGTCTTACCATGAGCAGATTGTATCCATTGGGCAA**ATAT**a**GGTTCGAT	−5.2
Zea	39,899–39,961	ATCGAACCCATAT**GTCGTCTTACCATGAGCAGATTGTATCCATTGGGCAA**ATAT**a**GGTTC**a**AT	−2.06
Lemna	41,245–41,307	ATC**a**AACCCATAT**GAAGTCTTCCCATGAGCAGATTGTATCCATTGGGCAA**ATAT**a**GGTTC**a**AT	−1.49
Acorus.ame	38,253–38,314	ATC**a**AACCCATAT**AAAGTCTTACCATGAGCGGATTGTATCCACTGGGCAA**ATAT**a**GGTTC**a**AT	−1.1
Acorus.cal	38,245–38,307	ATC**a**AACCCATAT**AAAGTCTTACCATGAGCGGATTGTATCCACTGGGCAA**ATAT**a**GGTTC**a**AT	−1.1
cPanax	40,104–40,166	ATCGAA**a**CCATAT**GAAGTTTTACCATGTGCGGATTGTATCCATTGAGCAA**ATAT**a**G**g**TTCGAT	
cNicotiana	39,789–39,851	ATCGAA**a**CCATAT**GAAGTTTTACCATGAGCGGATTGTATCCATTGAGCAA**ATAT**a**G**g**TTC**a**AT	

a The small inversions and SNPs in stem are marked with underlined bold capitals and bold lowercases, respectively.

b The length of small inversion is different from others.

c The sequence can not fold into stem-loop structure.

In addition, we detected 29 SIs when searching for IRs; nine SIs were confirmed and the rest were all putative because we could not find homologous sequences among other monocots (Table S2). They were mostly located in intergenic or intron regions and stem-loop forming. Excluding the previously-mentioned SI in *psaB*, we found three putative SIs in the coding region; one was known in *ccsA* [32]. If these putative SIs are proven common in the genome, they may provide phylogenetic information or may even play functional roles in stabilizing their corresponding mRNAs [32], [34].

### 6. Intravarietal single nucleotide polymorphisms (intraSNPs)

A plant cell often contains multiple clones or copies of cp genomes, and chloroplast can be regarded as a population with high genetic heterogeneity. Therefore, when thousands of high-quality cp sequence reads are aligned, polymorphic sites can be detected readily with software tools and confirmed experimentally. Similar phenomenon is also observed in mitochondrial genomes. The sequence variations within a variety (or cultivar and subspecies), often discovered by high-coverage sequencing, can be separated into major and minor intravarietal genotypes within a chloroplast or mitochondrial genome assembly based on sequence counts. Variations can be further defined based on experimentation over large population sampling from different varieties and subspecies of the same species as intervarietal or intersubspecific genotypes when one of the alleles become unique to certain faction of the samples [35], [36].

High throughput next-generation sequence technologies provide us a great opportunity to collect a huge number of raw reads for surveying intravarietal polymorphism within cp genomes. Using the 369,022 raw 454 cp reads, we surveyed each locus in date palm cp genome for possible intravarietal SNPs. A total of 113 SNPs were originally detected, and after an exclusion of homopolymer runs, the remaining 78 SNPs were considered to be stable SNPs. Among them, 16 fell into intergenic or intronic regions (Table S3) and the remaining were all located in protein-coding regions spreading over 23 genes (Table 4). There are 26 transitions and 52 transversions and more transversions are seen in both non-coding (93.8%) and coding (59.7%) regions. In the CDS regions, 29 and 31 mutations were synonymous and nonsynonymous substitutions, respectively. Major or minor genotypes occurred simultaneously in two SNPs of *rpoC2* at positions 21,213 and 21,215 in genome. In addition to these intravarietal SNPs, we also detected a 4-bp intravarietal Indel in intergenic region of *accD* and *psaI* from the position 61,482 to 61,485. This Indel, characterized as “TAGA”, however, is defined as a minor genotype, because in the total of 255 original reads (quality value greater than 20) in these four sites, only 75 displayed as “TAGA” insertion.

**Table 4:** The intravarietal SNPs found in coding region of date palm chloroplast genome.

NO.	Gene name	Major(minor) SNP type	Major(minor) Codon	Major(minor) amino acid	Major(minor) Reads number	Major(minor) reads percentage	Strand	Position in CDS	Position in genome
1	psbA^a^	C(T)	acC(acT)	Thr(Thr)	863(124)	87.4(12.6)	−	858	352
2	psbA^a^	G(A)	gGa(gAa)	Gly(Glu)	863(206)	80.7(19.3)	−	728	482
3	psbA^a^	A(G)	gcA(gcG)	Ala(Ala)	1167(296)	79.8(20.2)	−	564	646
4	psbA	T(G)	cTa(cGa)	Leu(Arg)	1117(250)	81.7(18.3)	−	350	860
5	psbA^a^	C(T)	tcC(tcT)	Ser(Ser)	1061(190)	84.8(15.2)	−	303	907
6	atpF^a^	A(G)	gaA(gaG)	Glu(Glu)	928(132)	87.5(12.5)	−	282	12699
7	atpI	A(T)	Agt(Tgt)	Ser(Cys)	774(93)	89.3(10.7)	−	559	15572
8	rps2^a^	G(A)	Gcg(Acg)	Ala(Thr)	558(62)	90.0(10.0)	−	475	16632
9	rpoC2	A(C)	aaA(aaC)	Lys(Asn)	564(211)	72.8(27.2)	−	711	20742
10	rpoC2	C(A)	caC(caA)	His(Gln)	988(202)	83.0(17.0)	−	636	20817
11	rpoC2^a^	T(C)	caT(caC)	His(His)	898(134)	87.0(13.0)	−	432	21021
12	rpoC2	A(C)	Aat(Cat)	Asn(His)	828(101)	89.1(10.9)	−	352	21101
13	rpoC2	T(G)	tTa(tGa)	Leu(−)	789(99)	88.9(11.1)	−	290	21163
14	rpoC2	T(C)	Ttt(GtC)	Phe(Val)	398(94)	80.9(19.1)	−	240	21213
15	rpoC2	T(G)						238	21215
16	rpoC1^a^	A(G)	aAt(aGt)	Asn(Ser)	624(231)	73.0(27.0)	−	1931	21750
17	rpoC1	A(C)	ttA(ttC)	Leu(Phe)	938(235)	80.0(20.0)	−	1503	22178
18	rpoC1	A(C)	ttA(ttC)	Leu(Phe)	702(225)	75.7(24.3)	−	1230	22451
19	rpoC1^a^	A(G)	ctA(ctG)	Leu(Leu)	952(223)	81.0(19.0)	−	1113	22568
20	rpoC1^a^	G(A)	gaG(gaA)	Glu(Glu)	762(181)	80.8(19.2)	−	1092	22589
21	rpoC1^a^	T(C)	ctT(ctC)	Leu(Leu)	674(231)	74.5(25.5)	−	885	22796
22	rpoC1	T(G)	ttT(ttG)	Phe(Leu)	256(53)	82.8(17.2)	−	537	23144
23	rpoC1	A(C)	Aga(Cga)	Arg(Arg)	529(171)	75.6(24.4)	−	199	24221
24	rpoB	T(G)	tTt(tGt)	Phe(Cys)	661(165)	80.0(20.0)	−	2744	24909
25	rpoB^a^	C(T)	tgC(tgT)	Cys(Cys)	748(137)	84.5(15.5)	−	2601	25052
26	rpoB	A(C)	tcA(tcC)	Ser(Ser)	319(114)	73.7(26.3)	−	2487	25166
27	rpoB	G(T)	Gat(Tat)	Asp(Tyr)	963(132)	87.9(12.1)	−	2035	25618
28	psaB^a^	T(C)	gaT(gaC)	Asp(Asp)	789(106)	88.2(11.8)	−	1092	39000
29	psaB^a^	G(T)	ggG(ggT)	Gly(Gly)	874(100)	89.7(10.3)	−	864	39228
30	psaB	G(T)	Gat(Tat)	Asp(Tyr)	868(106)	89.1(10.9)	−	814	39278
31	psaB^a^	A(G)	acA(acG)	Thr(Thr)	647(87)	88.1(11.9)	−	795	39297
32	psaB^a^	C(T)	agC(agT)	Ser(Ser)	861(109)	88.8(11.2)	−	720	39372
33	psaA	A(C)	ttA(ttC)	Leu(Phe)	521(210)	71.3(28.7)	−	2118	40252
34	psaA	A(C)	tcA(tcC)	Ser(Ser)	858(145)	85.5(14.5)	−	1800	40570
35	rps4^a^	A(C)	atA(atC)	Ile(Ile)	836(132)	86.4(15.6)	−	282	46306
36	ndhJ^a^	T(C)	ttT(ttC)	Phe(Phe)	407(113)	78.3(21.7)	−	456	49697
37	atpB	A(C)	gtA(gtC)	Val(Val)	1041(257)	80.2(19.8)	−	696	55445
38	accD	G(A)	gaG(gaA)	Glu(Glu)	708(88)	88.9(11.1)	+	48	59201
39	accD	C(T)	tcC(tcT)	Ser(Ser)	680(94)	87.9(12.1)	+	276	59429
40	accD	C(A)	Cat(Aat)	His(Asn)	863(108)	88.9(11.1)	+	403	59556
41	accD	T(G)	Tac(Gac)	Tyr(Asp)	852(106)	88.9(11.1)	+	406	59559
42	accD	T(G)	ttT(ttG)	Phe(Leu)	618(106)	85.4(14.6)	+	987	60140
43	accD	G(A)	gGa(gAa)	Gly(Glu)	877(114)	88.5(11.5)	+	1097	60250
44	accD	C(T)	tcC(tcT)	Ser(Ser)	848(102)	89.3(10.7)	+	1152	60305
45	accD	A(C)	Aaa(Caa)	Lys(Gln)	354(102)	77.6(22.4)	+	1177	60330
46	cemA	G(C)	gtG(gtC)	Val(Val)	1181(175)	87.1(12.9)	+	672	63407
47	petA	A(G)	gcA(gcG)	Ala(Ala)	844(113)	88.2(11.8)	+	828	64480
48	psbE^a^	G(A)	ccG(ccA)	Pro(Pro)	951(130)	88.0(12.0)	−	192	66208
49	psbB	A(C)	ttA(ttC)	Leu(Phe)	640(184)	77.7(22.3)	+	927	75429
50	psbT	A(C)	atA(atC)	Ile(Ile)	695(153)	82.0(18.0)	+	48	76250
51	psbT	A(C)	Aaa(Caa)	Lys(Gln)	233(151)	60.7(39.3)	+	100	76302
52	psbH	A(G)	acA(acG)	Thr(Thr)	782(115)	87.2(12.8)	+	120	76734
53	psbH	G(A)	ttG(ttA)	Leu(Leu)	651(89)	88.0(12.0)	+	174	76788
54	psbH	T(G)	atT(atG)	Ile(Met)	468(85)	84.6(15.4)	+	180	76794
55	psbH	T(G)	Tat(Gat)	Tyr(Asp)	469(85)	84.7(15.3)	+	181	76795
56	petB	T(A)	aTa(aAa)	Ile(Lys)	715(108)	86.9(13.1)	+	101	77843
57	petB	T(G)	Tat(Gat)	Tyr(Asp)	520(123)	80.9(19.1)	+	106	77848
58	petB	T(G)	Tac(Gac)	Tyr(Asp)	493(152)	76.4(23.6)	+	175	77917
59	petB	A(C)	gtA(gtC)	Val(Val)	576(219)	72.5(27.5)	+	450	78192
60	rps11	A(C)	Att(Ctt)	Ile(Leu)	1007(120)	89.4(10.6)	−	118	81436
61	ycf2	T(G)	ttT(ttG)	Phe(Leu)	917(121)	88.3(11.7)	+	3765	92696
62	ndhA	T(G)	tTa(tGa)	Leu(−)	793(100)	88.8(11.2)	−	1010	122904

a the minor genotype in date palm is major genotype in oil palm, or vice versa.

In general, SNPs in cp genome occurred at a rate of 1 in 1,400 bases. However, the 78 SNPs were not randomly distributed across the whole genome—a majority of these SNPs were clustered in LSC region. In IRs, we only found one SNP in *ycf2,* harboring a T–G mutation at position 92,696. In SSC region, we detected only two SNP sites: one in an intergenic region at 122,758 (T–G mutation) and the other in *ndhA* is similar to one locus in *rpoC2* (genome position 21163) with T–G mutation causing leucine (TTA) to a stop codon (TGA). The percentage of both minor genotypes in these two genes was about 11% with around 100 original supporting reads. These mutations occurred at 5′-end of *rpoC2* and 3′-end of *ndhA* could terminate the transcription prematurely and therefore make these two genes nonfunctional.

The intraSNPs in LSC region could be classified primarily into four functional gene categories: transcription (*ropB, rpoC1, and rpoC2*), photosynthesis (*psa* and *psb* proteins), energy metabolism (*atp*, *pet* and *ndh* proteins), and translation (*rps* proteins). Since chloroplasts can be considered a population within the cell, major-allelic variations that change amino acid sequences and translation efficiencies may affect or regulate gene functions. Furthermore, it has been proven in rice that the intravarietal major genotypes in one subspecies are often the minor genotypes in other subspecies, or *vice versa* [36]. Since subspecies among date palm cultivars are not yet well-characterized, we used the 81 available CDSs in oil palm cp genome for a brief comparative analysis [22]. We have adopted a procedure to avoid read contamination from nuclear or mitochondrial genomes and sequence artifacts (Materials and Methods); therefore, these intravarietal variations are unquestionably characteristic of the *Khalas* cultivar. Although date palm and oil palm belong to two different genera, we indeed found 19 sites, whose cp minor genotypes in date palm became major genotypes in oil palm, or *vice versa* (Table 4). This result confirms that some of these genotypes (major in one and minor in another) between the two species are actually real and these intravarietal cp genome variations may also be proven as real intervarietal variations among date palm cultivars if a large survey is to be carried out in the future.

Our study is the first one to quantify intravarietal polymorphisms using the next-generation sequencing technology. A recent analysis has reported a similar discovery of 40 SNP sites in *Loloum perenne* [37]. The intravarietal polymorphisms are indicators for the heterogeneous nature of chloroplast population in a given species and we have demonstrated in rice that the minor genotypes are also chloroplast in origin [36]. Since the effort of classifying date palm cultivars is early in its way, these intravarietal polymorphisms provide us useful markers for the evaluation of different subspecies. We are definitely able to validate some of these variations in the future genetic studies of date palm.

### 7. Gene expression analysis based on transcriptome data

We generated transcriptomic data, including 1,076,222, 833,875, and 465,456 raw reads for leaf, root, and bud, respectively (date not shown), for the cp transcriptome analysis. The genes identified from leaf were most abundant: 19,052 and 306,154 for protein-coding and RNA genes, respectively. Most of the genes (99) transcribed in at least two tissues, although we failed to detect transcripts for four genes (*trnQ-UUG*, *rpl32*, *ndhG*, and *ndhE*) in any tissues due to possible sampling biases (see discussion below). More than half of the 112 unique genes were rather scarcely detected in buds. The transcription-translation apparatus of chloroplasts has a number of prokaryote-like features; for instance, cp genes are often co-transcribed [38]. In our assembly, we found 18 polycistronic transcripts including genes for 63 proteins, 5 tRNAs, and all rRNAs (Table 5).

**Table 5:** Expression information of date palm cp genome in leaf, root, and bud.

Gene	Strand	Leaf	Root	Bud	Gene	Strand	Leaf	Root	Bud
psbA	N	44(100)	-	2(71.1)	***^a^***psaJ	P	76(100)	16(100)	1(88.9)
matK	N	107(100)	57(100)	2(100)	***^a^***rpl33	P	41(100)	24(100)	-
rps16	N	122(100)	120(100)	105(100)	***^a^***rps18	P	10(100)	2(100)	-
***^a^***psbK	P	6(100)	-	-	***^b^***rpl20	N	35(100)	34(100)	-
***^a^***psbI	P	13(100)	-	-	***^b^***rps12_5end	N	20(100)	15(100)	2(100)
***^b^***atpA	N	143(100)	28(100)	6(100)	***^b^***clpP	N	151(100)	174(100)	18(100)
***^b^***atpF	N	15121(100)	3030(100)	91(100)	***^a^***psbB	P	34(100)	5(100)	6(100)
***^b^***atpH	N	96(100)	20(100)	7(100)	***^a^***psbT	P	14(100)	-	-
***^b^***atpI	N	67(100)	10(100)	6(100)	***^a^***psbN	N	37(100)	-	-
***^b^***rps2	N	14(100)	6(100)	2(64.7)	***^a^***psbH	P	73(100)	2(100)	-
***^a^***rpoC2	N	30(100)	29(99.5)	4(91.1)	***^a^***petB	P	19(100)	2(77.5)	2(62.4)
***^a^***rpoC1	N	84(96.5)	63(100)	9(98.3)	***^a^***petD	P	12(100)	5(98.7)	-
***^a^***rpoB	N	59(100)	47(100)	5(64)	***^b^***rpoA	N	61(100)	21(100)	3(100)
petN	P	5(100)	5(100)	2(100)	***^b^***rps11	N	121(100)	15(100)	-
psbM	N	5(100)	2(100)	2(100)	***^b^***rpl36	N	8(100)	2(100)	1(100)
***^b^***psbD	P	165(100)	5(72.5)	2(90.7)	***^b^***infA	N	30(100)	10(100)	-
***^b^***psbC	P	140(100)	5(78.2)	-	***^b^***rps8	N	24(100)	8(100)	-
***^b^***psbZ	P	233(100)	19(100)	2(100)	***^b^***rpl14	N	43(100)	30(100)	3(100)
***^a^***rps14	N	39(100)	-	-	***^b^***rpl16	N	14(100)	8(97.8)	3(100)
***^a^***psaB	N	150(100)	16(100)	-	***^b^***rps3	N	40(100)	24(100)	4(100)
***^a^***psaA	N	29(100)	7(100)	2(68.7)	***^b^***rpl22	N	40(100)	18(100)	4(100)
ycf3	N	268(100)	177(100)	4(78.9)	***^b^***rps19	N	19(90.3)	8(90.3)	5(90.3)
rps4	N	5(84.8)	1(84.7)	-	***^b^***rpl2	N	238(100)	122(100)	7(100)
***^b^***ndhJ	N	19(100)	8(100)	3(100)	***^b^***rpl23	N	19(100)	3(97.9)	1(100)
***^b^***ndhK	N	9(100)	4(97.7)	2(74.9)	ycf2	P/N	17(100)	35(84.3)	16(97.8)
***^b^***ndhC	N	13(100)	4(100)	-	ndhB	N/P	25(88.5)	14(70.7)	-
***^a^***atpE	N	144(100)	20(100)	4(100)	***^a^***rps7	N/P	317(100)	85(100)	11(100)
***^a^***atpB	N	114(81.7)	14(87.4)	7(100)	***^a^***rps12_3end	N/P	126(100)	32(100)	9(100)
rbcL	P	158(100)	12(100)	9(95.4)	ndhF	N	2(66.1)	5(60.9)	-
accD	P	25(100)	47(100)	12(99.9)	rpl32	P	-	-	-
***^b^***psaI	P	1(82)	1(82)	-	ccsA	P	2(98)	4(97.7)	2(91.5)
***^b^***ycf4	P	64(100)	12(100)	3(100)	***^b^***ndhD	N	17(72.5)	-	5(100)
***^b^***cemA	P	7(97.6)	7(97.6)	-	***^b^***psaC	N	5(100)	3(100)	-
***^b^***petA	P	50(100)	17(100)	2(50.8)	ndhE	N	-	-	-
***^a^***psbJ	N	8(100)	3(100)	1(97.6)	ndhG	N	-	-	-
***^a^***psbL	N	10(100)	4(100)	1(100)	***^a^***ndhI	N	8(100)	4(98.7)	-
***^a^***psbF	N	8(100)	1(100)	-	***^a^***ndhA	N	21(100)	6(100)	-
***^a^***psbE	N	11(100)	1(100)	-	***^a^***ndhH	N	18(100)	6(100)	-
***^b^***petL	P	1(76)	1(100)	-	***^a^***rps15	N	4(87.9)	1(95.2)	-
***^b^***petG	P	7(100)	3(100)	-	***^a^***ycf1	N	33(100)	32(100)	14(100)

1. the numbers outside the parenthesis mean the number of original reads and inside mean the percentage of the gene covered by all related reads (-, no supporting reads).

2. Each continuous bold and italics “a” or “b” superscript at the outset of genes means one polycistronic transcription units.

3. N, negative strand; P, positive strand.

Since the cDNA identification for chloroplast genome is not as quantitative as it should be due to the lack of polyA tail that provide a handle for mRNA purification, the interpretation of cp gene transcription is rather qualitative. What we observed here is the transcripts whose RNAs are co-purified with mRNA preparation, similar to rRNAs, as they both are so abundant. It is also the unbiased co-purification that provides us useful information about chloroplast transcripts. We indeed observed interesting expressions of the date palm cp transcripts. First, one protein-coding gene—*atpF*—have 15,121, 3,030, and 91 corresponding cDNA reads, accounting for 79.4%, 66.8%, and 21.9% of the total cp related reads in leaf, root, and bud, respectively. Although the fractions may reflect the real proportions of the gene abundance in three different tissues, its unusual abundance is obvious by examining the assembly—its sequence has a polyA-like track that is 22 bp long and situated at the 57-bp upstream of *atpF* gene. Since our reverse transcription was primed by using a polyT adaptor, *atpF* transcripts are thus enriched in the cDNA libraries. However, due to the prokaryote-like features of chloroplast genome, there is no obvious polyA structure at the downstream of other genes. Furthermore, we found that polycistronic transcript *rps2-atpI-atpH-atpF-atpA-trnR-trnG* had only 50 transcripts containing *atpF* and *atpA* and it is obvious a result of enrichment related to the polyA-like track (Figure S2). In fact, other than *atpF*, the fraction of total transcripts with adaptor sequences account for about 72% of all CDS reads, and it indicated that our transcripts for CDS are indeed non-specifically co-purified with polyA mRNAs rather than enriched due to polyT affinity purification. For example, in the downstream of *atpA*, there is an 11-bp (10,718-10,728 in genome) rather degenerated polyA unit, whereas only 23 reads with adaptors for *atpA* were detected. Besides, the extremely high number of *atpF* transcripts suggested that this gene may not be simply co-transcribed with other genes as a polycistronic transcript but may also be transcribed independently. Second, there are two rRNA genes—*rrn23* and *rrn16*—also display high abundance. In leaf, the number for *rrn23* and *rrn16* are 280,106 and 28,721, respectively (Table 5). There is no polyA structure downstream of these RNA genes, and therefore their transcripts may be just co-purified with mRNA but such enrichment may reflect their real abundance in leaf. Third, the number of *trnA-UGC* transcript is also special, accounting for 90.9% of all tRNA transcripts. It is curious why this gene had so high a copy number in the cell, since alanine only accounts for 5.32% amino acids in date palm cp genome. The reason remains to be illuminated.

In summary, we present here the first complete cp genome from Arecaceae family obtained based on pyrosequencing. The genome is A+T rich and we had to design primers to verify the most of the homopolymer runs. As a rather regular cp genome, its gene content and structure are similar to those of tobacco, and its IRs expansion occurred in LSC but not SSC regions. Its number of repeated sequences appeared lower than that of Poaceae cp genomes and certain dicots. The high coverage of raw cp reads demonstrated that the intravarietal SNPs exist not only in intergenic region, but also in coding genes, where these variations may have been functionally selected. Some of the major intervarietal SNPs may serve as genetic markers for classifying subspecies when appropriately developed as simple genotyping assays. Most protein-coding genes of cp genome are transcribed as polycistronic transcripts and transcriptome analysis is to be designed in particular to avoid cross-contamination between the nuclear poyA-containing and organellar polyA-lacking transcripts. Our data are useful for the study of date palm biology and pave a way for further molecular investigations.

## Materials and Methods

### DNA sequencing, genome assembly, and PCR-based assembly validation

We collected fresh green leaves from an adult plant of *Khalas* (a common cultivar of date palm) grown in Al-Hass Oasis, Kingdom of Saudi Arabia, and extracted genomic DNA from 50 g leaves according to a CTAB-based protocol [39]. We used 5 µg purified DNA for constructing libraries according to the manufacturer's manual for GS FLX Titanium.

Since our original sequence reads are a mixture of DNA from nucleus and organelles, we separated the cp reads from the total reads based on the known cp genome sequences. We assembled the filtered sequence reads into non-redundant contigs using Newbler, a *de novo* sequence assembly software, and aligned the contigs to the references, including those of tobacco [5], rice [40], orchid [8], and the coding sequences from oil palm [22]. We obtained 42 cp genome contigs with a collective length of 115 kb. Using a perl script, we made an iterative elongation for both ends of each contig against the total raw reads until the 42 contigs emerged as preliminary cp sequence scaffolds.

To avoid assembly errors from homopolymer runs (characteristics of the pyrosequencing technology) and to acquire a high-quality complete cp genome sequence, we designed 151 pairs of primers covering the preliminary cp genome assembly. Based on a published procedure [41], we sequenced PCR products using BigDyeV3.1 Terminator Kit for 3730XL (Life Technologies) and assembled the high-quality sequences into the complete cp genome using the Phred-Phrap-Consed package [42], [43]. The four junctions between the single-copy segments and inverted repeats were also confirmed based on PCR product sequencing. The final date palm chloroplast sequence has been deposited to GenBank (accession number GU811709).

### Genome features, gene content and codon usage

The genome was annotated by using DOGMA [44], coupled with manual corrections for start and stop codons. Intron positions were determined based on those of the tobacco cp genome [20]. The transfer RNA genes were identified by using DOGMA and tRNAscan-SE (version 1.23) [45]. Certain intron-containing genes (i.e., *trnK-UUU*, *petB*, and *petD*), in which exons are too short to be detected with software tools, were identified based on comparisons to corresponding exons in tobacco and other cp genomes. The functional classification of cp genes was referred to CpBase (http://chloroplast.ocean.washington.edu/). The *cemA* sequences of various species were downloaded from Genbank and aligned by using MUSCLE [46]

### Repeat structure and small inversion

We used REPuter [47] to assess both direct (forward) and inverted (palindrome) repeats within the date palm cp assembly. The identity and the size of the repeats were limited to no less than 90% (hamming distance equal to 3) and 30 bp in unit length, respectively. Verification of the identified repeats was performed manually based on intragenomic comparisons.

To obtain possible small inversions, we first searched IRs in the length from 11 to 24 bp with REPtuter, and then collected those repeats whose distance is shorter than 50 bp as candidate SIs. Next, we evaluated the likely secondary structure of these SIs using MFOLD (version3.2) [48], and discarded those do not form obvious step-loop structure. Finally, we run Blast program using the remaining putative SIs against all monocot cp genomes, and only collected those qualified hits into the final SI list.

### Intravarietal single nucleotide polymorphisms

We used the total 369,022 cp reads for intravarietal SNP discovery. Based on the Consed graphical interface, we were able to identify genome loci that have more than one nucleotide type as candidates for intravarietal SNPs and to assign major and minor alleles. We only considered those loci with nucleotide sequencing quality value greater than 20.

To make our results more convincing and to eliminate false positives caused by contamination from nuclear and mitochondrial sequences, we adopted the following measures: (1) the number of aligned reads for each selected locus must be above 50; (2) the percentage of the maximal minor genotype (if there are two or more minor genotypes) must be above 10%; (3) if there were gaps in the reads of one aligned locus, we did not count (it may be a one-basepair Indel), and after removing these reads, the number of the remaining reads must be more than 50; and (4) we excluded the sites overlapping with homopolymer runs. These errors often occurred at two ends of a homopolymer run due to high coverage of the original reads; for example, a sequence “AAAAAAAAAC” often displays a minor genotype for C in position 10, but this C is often an artifact due to sequence errors intrinsic to pyrosequencing.

### Transcriptome analysis

We purified total RNAs from leaf, root, and flower bud of cultivar *Khalas* and sequenced their cDNAs using Roche GS FLX system according to the manufacture's protocol. The total RNA was reverse-transcribed by using an oligo-dT adaptor. The adaptor sequences are:

5′-ATTCTAGAGACCGAGGCGGCCGACATGTTTTGTCTTTTGTTCTGTTTCTTTT-3′

After reverse-transcription, the cDNAs were sheared into small fragment to construct sequencing libraries followed by emulsion PCR and sequencing. We mapped all raw reads to the date palm cp genome assembly using both Newbler and Blast programs. We acquired non-redundant contigs and singlets by assembling the mapped reads, and collected only those with over 80% coverage and 99% identity of a gene. If different genes or a single gene are assembled into one contig or singlet, we assign the genes as polycistronic or monocistronic transcription unit, respectively. The poly- or monocistronic transcripts were estimated using the assembling results from leaf where chloroplasts are most abundant. These transcription units are carefully inspected manually using Consed graphical interface to avoid assembling mistakes and interrupting contigs by intron-containing genes.

## Supporting Information

Table S1 A whole list of all pairs of primers used in date palm chloroplast
(1.08 MB PDF)

Table S2 The location and sequences of all putative small inversions in date palm chloroplast genome
(0.01 MB PDF)

Table S3 The intravarietal SNPs found in non-coding regions
(0.01 MB PDF)

Figure S1 Multiple alignments of cemA genes from 77 different cp genomes.
(0.02 MB PDF)

Figure S2 Partial 454 transcription reads alignments with date palm cp genome at downstream of atpF and upstream of atpA. Comments are marked with thick blue line and blue color fonts.
(0.25 MB TIF)
