# Supplementary material for: The Complete Chloroplast Genome Sequence of Date Palm (Phoenix dactylifera L.)
Source: PLoS One. 2010 Sep 15;5(9):e12762. doi: 10.1371/journal.pone.0012762 (PMC2939885; doi:10.1371/journal.pone.0012762)
Supplement: Table S1 — A whole list of all pairs of primers used in date palm chloroplast (1.08 MB PDF) [file pone.0012762.s001.pdf]

Table S1. A whole list of all pairs of primers used in date palm chloroplast.

1F	TATTGCTCCAATCCTTCAACG	1R	TACATTGGATGGTTCGGTGTT
2F	CTCATAAGGACCGCCATTGTA	2R	TCATTATCACAGTGGATCCTCAA
3F	CGTTCTATATGCGATTCATGT	3R	TCCATTCTTGCTGCGATTAGT
4F	CTTGGATCCAGAATTGAAGGA	4R	ATAGATTAGTGCCTGATGCGG
5F	TGGTCAGAACGGGGTATGT	5R	GCCGGATTTACTGGCATAC
6F	CATAGTCGCACTTAAAAGCC	6R	GAAGGAAGAGATCTCAGAAAAGT
7F	CCTTAAAATATCACAAACAGTTCC	7R	ACCGGTTGAGCCAATGACTAT
8F	GCCAAGAACATCTTCATTCCT	8R	TTCACATTCAGGATATGCTCGT
9F	TCCACATAGACTGTGGAATTGG	9R	CACTGTAAGGTGAGAGCGAGG
10F	TTGGCAAGCCGCTGTAAGT	10R	AAGAATCATTGAAGCTGGATCA
11F	AACCCTCGGTACGAATAACT	11R	AAAATTATGCTTCGCGACT
12F	GGACGAAGTGTCAACGCTA	12R	TTAGCTTGGAAGGCTAGGG
13F	GATATGGAGTCGCGAAGCA	13R	TTAGACAATGGACGCGTTT
14F	CGACTATAACCCCTAGCCTT	14R	AGTAGGATCCGCAGCTCAAA
15F	GCTTCTAACTCCGCGAATTG	15R	GATGGCATTGCTCGTATTCAT
16F	CTCACTCACAGGTATCTGAGCAA	16R	TCTGGTCGGAAGAGTCCTTCT
17F	TGGCCCCTCTACGTAATTC	17R	CATGAGACCCAAGACTTAACC
18F	TCGTTGAGATCGTGCACTT	18R	CGTTATTGCTGCTGGATTG
19F	TTCTGGTTGTCTCGCAATAC	19R	CCCAATACTTTTACCAATTAACAT
20F	CATGGATTCACCTATATAAGCC	20R	CAGCGGATTCAGTAGCATCAG
21F	CGATGATCACAATATCAGGCA	21R	TGGACGAAGCGATCTGTTATC
22F	TGAATGTTCTTGTCTTGCCTTG	22R	CCAATTGTTCTCGAATTGGTC
23F	GGGATCGGGATTATAAATTC	23R	AAATACCAGAAGGTAGATCGG
24F	CGCTGTGCCTGGATATCTTAT	24R	GTAATTGCTGAGATTCGTGCC
25F	CGCGGACCTCCTGCTAATA	25R	TCCTTCTAATCCAGTCCATCTGA
26F	AGGTATCCGGCATCTGATGTT	26R	AGATCAACGTGTCATTGGCTC
27F	CAATCTCAATCTTGATTCACG	27R	CCAGGGGAATTAGTAATGTGT
28F	CGAATAATCGACCCGTTTAC	28R	GCCTGGTATACTGCGATGTGT
29F	ATCGACCATGGATCAATTCC	29R	CGCACTTGTTACACAACAACC
30F	TAGTTCTCGAACGAGCAATCG	30R	AGACTCATGTGACAAGCCAGG
31F	TGGAGACCTGAATGCCAAGTA	31R	TCATGTGAGGATTGGTCATTG
32F	GAATTCTTGACGATATCGAGCTG	32R	CGTTCAGAATTGGACAATAACG
33F	TTGTTTCCTACTCACGCGG	33R	AAATAGATTTCCGGTCACAGA
34F	CAACAGAAGTCAGGTCGCACT	34R	GTGGAATCAATGGTGCAGAGT
35F	CATGTTCCATTAGGAGCATTCC	35R	TGGCTGGAATCATAAGTATCTGA
36F	AAAAGAGTTGGCCTTGGAT	36R	CTCATGCAAATTGCACACT
37F	GGTCACACAGATCCAAACAGT	37R	GCCCCTATCGTCTAGTGGT
38F	GATGTCCTGAACCACTAGACGA	38R	TTCGTTCTCTATTGCATTGGC
39F	CTTAGACCACGCAAGGCAAG	39R	AACAGCAATTGGAGCAGAGAA
40F	GCCAGTTCCTACTTGGAAGGT	40R	CAGAATACGATTAATCCGGCA
41F	CGTGACCAAGAAACCACTG	41R	AAAGACATACAACCTTGGCA
42F	TTTTGGGATCTTCGTGCTC	42R	TGTACCCGTTGTATTTGCTT
43F	TTGGAGGATTACAAACATGAC	43R	ATACAGAATGGTAAAGAGGGC
44F	GAACTGGGAACCAATGGAC	44R	TAACATCCAGAAAACTGCCA
45F	CTCTTAACCATCCCATCAAAT	45R	ATAGCTCCATGAGCAAAGG
46F	TGGCCCAACTTAAATGAGA	46R	TGGATACAGGACCCTTTACA

47F	TGATACTGTGTCCAATTCCGA	47R	TGAGCATTGTACAAGGACGTG
48F	GAAGTCATGTGCGGTAGCAAT	48R	TTCTCATAGTTGGTGCTGCTG
49F	TGCACCAGGAGCTGTTACC	49R	GGATCTGGAACCTACATGCT
50F	TAGTTATTCCAGATGCTCGCC	50R	AATTGGTTGAAGATCACGAGG
51F	ATTGATGGATCCAACACCC	51R	AATAATATGGCCGTGATCTG
52F	TTGGTTCTCGTGAAGTCTCTT	52R	CCTCGGATGCTTCAATTGTTA
53F	AAGTACGAACGAGATGGATGTT	53R	ACAGGTATGACCGATCAATGG
54F	CGTTTTGTTTACATGACGTG	54R	TCGCAATATCGTATTCGTCTA
55F	GGCTTTTCCAGCGATATGTA	55R	ATAGCTAATAGCCCTAAGCTAAGAT
56F	ACATTTTGAGAAAAGGAGAGATTA	56R	TCTACCAATTTCGCCATATC
57F	TTATAGCGTATAGCGAGCGGA	57R	TGTCGGTATTGACATGTAGAACG
58F	TTCAGAGAAACCCTGGAATTA	58R	GTCTGAAATGCATCCTGTCTA
59F	AAGGATAGGAATAGACAGGATGC	59R	CGAATTAGTGAATCAGGCAGG
60F	ATGGAGTACCAATCCTCGGTT	60R	GATGCTTGGTTACAATTCCGA
61F	GACCATTCCAATGCTCCTT	61R	AACTCGCAACTCAACGGATT
62F	TCTAAAGCACCGAACCATTA	62R	ATCTAGGCCGAAGGCAGAC
63F	GGTCATATGTAATCCATCCACG	63R	CGGATCAGAGAGTTATCATGTGG
64F	TCAGGCTATTGTTCTCTTGTTCC	64R	CGGAGAATTAGACGGTCTTCC
65F	CCTATATCTACAGCCGTGGCA	65R	AGTAGGCGAACGTACTCGTGA
66F	AGGCGGATACTTCTGATCCTG	66R	CGATCAACTTGCTATCGGACA
67F	GCTGACCGACAGTATCTCGAC	67R	CTCGATATGGATTAGGCGTGA
68F	CCGAACCATTTTAACATAACA	68R	TGGTATGCATTTTCGTGTACT
69F	TCATTATTGCCGCGATAAC	69R	GTCTTGATGCTATTGGGCA
70F	TCAATTCGATGTTGTCTAACG	70R	AATGCAATGTAGGCATTAGG
71F	AATGGCTAAAATATCTTCCGCT	71R	GGTGCCTCGATTTAATATTTC
72F	GCGAATTATCATACATATTCGTG	72R	TTTGTTCAATTTCTCGTGGA
73F	GGAAATTAGAGGCCAAGGAG	73R	TGACAAGTTCTTCATTACCCAG
74F	AATTGGTCAAAACACACAATG	74R	AACAGATGCCAAGAAGAACAA
75F	GATGTATCGGATGGACATCAAG	75R	TCTACGGATGAGACATCCTGC
76F	ATTACTATTCCTTGCTTTCCG	76R	GTTCACGGATTAGCTGTACCT
77F	TTTCATCAAGTGGTCATGC	77R	CAACCGTTGTTGCATCAC
78F	ACATTTCAATTATATGCGAAATC	78R	GCGCTCTTAGTTCAGTTCGGT
79F	AGACGTGGTGATCAGTTGGAC	79R	ACGTCACGTGGAATTGTATCC
80F	TCTTGAACTACAACATCTGACCA	80R	TTGATTCGCATCGTTTATATTA
81F	CAATACGTCCAGTCGATTAGAAT	81R	ATACAGCTCGGAAACGTCG
82F	CGAGTGATCCACAAACTACG	82R	ACATGCCAACTATTAAACAACTTA
83F	TTTCTTATTGGCTGTCTTGTG	83R	TAGCATTCCCTCACGCTTG
84F	TTGCTCATATCGAACTCGAAG	84R	AACTGCCAACCAATGGAGTAG
85F	TTGAACTAACCTCTCATTGATGT	85R	ATGAACACGATACCAAGGCAA
86F	CATTGCGTATTGGTACTTATCGG	86R	TGTATAGACGTTGAGGCGGAC
87F	TTGTATGGTCCTGGAATATGG	87R	AATCTGGATCAATACCAGCAA
88F	GTTCTATGGTGGCGAACTCAA	88R	CCATATTCCGAATTCAATGGTT
89F	AACCAGATATGGAGATGGCG	89R	ATCGGTACAGGCCTCTTGAAT
90F	TGTTGAGTCATTACATCCATTCA	90R	TACGGCAGTAAGAAGAGGCAA
91F	AACTGGTTATTCCTTACCTCGG	91R	CGAAGGAACCGGACATGATA
92F	AGTTGCAACTCCGTAAAATG	92R	CGACACAACATATAGCTAACCTG

93F	GTGCTTCCTTAGGAGTTAAACTT	93R	TCGTTATGATTCAACCAGAGGA
94F	ACTATAATTCGTCCGCGCCTA	94R	TTAATAGATTGTGTCTCACGCA
95F	GATGCTATTCGAACTGTTCCTT	95R	CGGTTCTGCAATAGAGATGGA
96F	CCGCTTCTATCTGTCTAGCTGT	96R	TTGCTTCGTATTGTCAAGATCC
97F	TTTATCACACTGCCTTTTATGAG	97R	ACAACCCAAAGTCACCGT
[1]98F	GTCTCCGGTTCACAGAATGAA	98R	AATTCGCGCCTACTCTGACTT
[1]99F	CACCAATGCCAATGAATGATA	99R	GCTTAGCAACAGTCGGACAAG
100F	TACTTCTTCTTCCAAGCGCAG	100R	AGTAACGGCAGCAAGTGATTG
101F	CGTCGATACTTCCACATATTACG	101R	TCAATGGAATCTCATCATCCA
102F	CCTAGTTGATCCTGATTCGACA	102R	GAGATCTCAATTCCAGCTACGG
103F	TTCGCTCCTGTATCTTCCAA	103R	TTCTCACGTTCCGTGAATAGC
104F	AAGGATCTTCTGATCAATCCA	104R	AGTTGCGATCGGATCTATTCA
105F	ATCGGTCCGGATCTACTGC	105R	TGGATCATTCAGGAATCGAAG
106F	TCTTGATCAATGGAGGAACAA	106R	TCCAGCAACAAGAAGAAGGAA
107F	ATGGCCAATTCCTCAGAAGAT	107R	TTAGGCTTGTCATCCAGGAAC
108F	AAGACGGATATTCCTTGCTCA	108R	CTTCATGCTCGTTCCAAGTTC
109F	GCTGTTGCACAAAATGTACTT	109R	GCCACATATCTTTACGGCTA
110F	CCTATCTAACGGAACGCTATT	110R	GAATAGGCGTAATCGGACCTG
111F	TTCTATCGATCGGTCATGTCA	111R	ATGCTTGCATATTCGTCCATC
112F	TCCTGCATAATCTCGAATGTT	112R	GACATATACGAAGGAGTGCGG
113F	AAGAGATTCGTTGTTCCTGACC	113R	TCGGAGATTGGATGCAGTTAC
114F	TCTCTCCATCGGAACAATAGG	114R	ACGTCGAGGTACTGCAGAAGA
115F	ATGAGTCGATCCGCCTACAC	115R	ACGAGGAGCCATATGAGGTG
116F	GGTTCCTCGAACAATGTGATA	116R	TAACCGCATGGATAAGCTCAC
117F	CCCATAGGTATAGCGTTTGA	117R	CGAGAAACTCAACGCCACTA
118F	TCGAATAGAACATGCTGAACA	118R	CAATCATTCCGGATAACGC
119F	GGTGAGGCAATAGCTTACCA	119R	GGCCCAACCCTAGACACTC
120F	CTTCCCGAAAGGAAGAACG	120R	GACTCCAACTATCGTCCATGT
121F	GAACAGGCACACTTGAAGAGC	121R	TTCTACCATAGAGGCCAACGA
122F	AACCAGCATTCTTAAGACCAA	122R	GCGGTCAGAGATCACATTCTC
123F	CATCACTAGCTTACGCTCTGACC	123R	CGTTACTCAAGCCGACATTCT
124F	TACCAAATCGAGGCAAACT	124R	CCCTTCTCCCGAAGTTACG
125F	TCTAAGGAACTCGGCAAAATA	125R	TCTAAGTAGTAAGCCCACCCC
126F	TATCGTGCCCACGGTAAAC	126R	GTCGAAATGGAATGGCCTT
127F	ACCAATCCATCCCGAACTT	127R	TAATAAATTCGGTCGTTGTGG
128F	GTTCAACTCCCGTTCTCAA	128R	AACAACTTCCGAAACGAAG
[2]129F	CAATGCGTAATCTCAGCATTC	129R	ATAATTCTCTTAGGGCTCCCT
130F	CCCATGACTAAAACCCTAGTA	130R	TCTTATCGAGCGGCTTTAT
131F	TTCTGGTGAATATCCAACAATAG	131R	GACTTATACTTATTCCGAAAGCAA
132F	AAACCCCATAGCTATACTTAACAG	132R	TTTTCATTATTATTGTTTATTGTT
133F	GTCCAAATCCTATTGACATAGTAAC	133R	CAGTTGAAAGACCCGACG
134F	TATGATATTTGTGACTTTAGA	134R	TGCTTCATTAGCCCATACTG
135F	TGGATTATTGGAGTTATCGTATG	135R	TTGGTAATAGTTGGAACGATGC
136F	CGTATTCTATCACAACTCGTTCC	136R	CAATTGGACGATCCATTAATTC
137F	TGACCGCTGTACATTGCTAAC	137R	ACATGTGCAACTCGCATGAT
138F	GGCTCGTACACATTGAGTACA	138R	GGAGATGGGGTTACTTCACTG

139F	CGAGCCATAGTAATTGCACCT	139R	CCGGGTTCATGAATTATAGTC
140F	ACGGAAACGTTCTGATGTG	140R	TCCCTACGATTCCGATCTC
141F	GTTCGGTATTTTCGCCCTC	141R	GGGTACCGCCTTGTTTTAG
142F	GCTCGTAGACCACCTGAAA	142R	AATGGGATCTTCGTAAGGTT
143F	AAAGCGTCGGACTTCTAAAT	143R	AATAATTCTGGGAAAACGTCA
144F	GATCTTTTCTTGTAGCCGGT	144R	TGGCAATCGTTTCAGTATGT
145F	AATTGAGTTGATACGCCATTAG	145R	AAAATGTTGAAGACAATTACGC
146F	TGAATCGAGGATATAGCAATTTT	146R	CCGAATCATTTTATGTATTGGTAA
147F	GGTCCAGGTCTCAATGTCG	147R	ACGGAAACATATTTTGCAGA
148F	CGATTTGTTTCATCGTTGTAA	148R	AGGATTTGAAGGAATGGAATA
149F	TCGCCAAGGTTTAAGAGAG	149R	TTTATTGAGCGGACCATGC
[3] 150F	CAAGCGAATTCTTTTGATGTT	150R	TCTTAGTGACAATAGGCGACG
[4] 151F	GCTGTGACACGTTCACTAAAA	151R	GGTCGTGTTATCAACACTTGG

[1], Primers used to verify JLB
[2], Primers used to verify JSB
[3], Primers used to verify JSA
[4], Primers used to verify JLA
