# Supplementary material for: The Complete Chloroplast Genome Sequence of Date Palm (Phoenix dactylifera L.)
Source: PLoS One. 2010 Sep 15;5(9):e12762. doi: 10.1371/journal.pone.0012762 (PMC2939885; doi:10.1371/journal.pone.0012762)
Supplement: Table S2 — The location and sequences of all putative small inversions in date palm chloroplast genome (0.01 MB PDF) [file pone.0012762.s002.pdf]

Table S2. The location and sequences of all putative small inversions in date palm chloroplast genome.

No.	Gene location	Genome coordination	[b]Sequence alignment	Free energy (ΔG)
1	[a]rps19(3')-(3')psbA	69-111	TGGAGCAATACCCAA**CTAAAAGAAGATA**TTGGGTATTGCTCCA	-15.57
2	trnK intron	3,830-3,872	AATTTTTTTTTT**ca**CTT**CCAAACAAA**AAG**aa**AAAAAAAAAATT	-7.81
3	trnS(5')-(5')trnG	8,572-8,602	TTATTTATTTAT**AATTATA**ATAAATAAATAA	-5.63
4	trnS(5')-(5')trnG	8,744-8,808	ATATTATAATGTT**t**ATTTATATA**ACAGTTTATTTATATAACA**TATATAAAT**g**AACATTATAATAT	-12.45
5	atpF intron	13,190-13,238	CTATTTTTTTT**TTACGTAGGTCGTCGATTCGGCATTGG**AAAAAAAATAG	-5.42
6	atpH(5')-(3')atpI	14,566-14,636	AAAAAAAAAATG**g**T**t**AA**TGATACAATCAACCAATGAATTTTTACTTAATTTTTT**TT**t**A**t**CATTTTTTTTTT	-6
7	atpH(5')-(3')atpI	14,998-15,046	TAATATATATATAT**ATAGGGTAAGGGTATATAATA**ATATATATATATTA	-5.72
8	trnE(5')-(5')trnT	32,029-32,061	ATCATACTATGAT**AATCATC**ATCATAGTATGAT	-8.95
9	[a]psbC(3')-(3')trnS	35,898-35,926	TGGCTCGGCTA**TCCCACC**TAGCCGAGCCA	-12.2
10	trnG(3')-(3')trnfM	37,053-37,104	AACTACTATACTAACTACTATA**CTAACTAC**TATAGTAGTTAGTATAGTAGTT	-17.27
11	trnT(5')-(5')trnL	47,138-47,178	TCTATATTATTAGTTATA**ACTAA**TATAACTAATAATATAGA	-12.9
12	trnT(5')-(5')trnL	47,397-47,440	TTAATATATATATATAT**g**TT**AATG**AA**t**ATGTATATATATATTAA	-7.71
13	[a]trnM(3')-(3')atpE	54,067-54,108	ACTTATTAGATA**CCGAAGTCAATTGAGTGA**TATCTAATAAGT	-6.4
14	atpB(5')-(5')rbcL	59,678-59,718	CAGTTACATTT**at**AGT**TTCATTTGT**ACT**ga**AAATGTAACTG	-7.95
15	accD(3')-(5')psaI	60,797-60,858	TTTTTTATCCT**ACCTCTATTCATGATTAGTAATCACAAACCTTCTATCAAC**AGGATAAAAAA	-6.66
16	accD(3')-(5')psaI	61,382-61,409	TATATTTATAA**ATAATA**TTATAAATATA	-5.22
17	[a]petA(3')-(3')psbJ	64,885-64,924	CGACACAAGAAAAGGG**ATTTTCCA**CCCTTTTCTTGTGTCG	-17.49
18	[a]petA(3')-(3')psbJ	65,533-65,597	AGTAAGAACTCAGCGGGACC**CTCCTTTGTTTGATTAGAGCAGTAA**GGTCCCGCTGAGTTCTTACT	-22.53
19	clpP intron	73,230-73,275	AAAAAAAAGAA**TCAATGTGTCGATTCCAGTTCTAT**TTCTTTTTTTT	-5.33
20	[a]psbT(3')-(3')psbN	76,332-76,374	TTGAAGTAATGAGC**c**TCCCA**ATA**TGGGAG**a**CTCATTACTTCAA	-16.79
21	rps8	82,300-82,357	AATTCTTTCTA**GTCGAGCTTCTCGATCTGTCATTATACCTCGAGAAG**TAGAAAGAATT	-12.35
22	rpl22(5')-(5')rps19	86,257-86,301	TTAATAATATTT**CTATTAATATTGAATATTCAA**AAATATTATTAA	-6.48
23	[a]trnL(5')-(3')ndhB	97,362-97,410	ATGAACTTATAATC**TGATGATCGAGTCGATTCCAT**GATTATAAGTTCAT	-10.97
24	ndhB intron	98,353-98,388	AAAAGAAAGAAGA**ACTGAACTCA**TCTTCTTTCTTTT	-10.09
25	rpl32(3')-(5')trnL	116,315-116,354	TTCACAATAGAAAA**ATAGGATTAAGA**TTTTCTATTGTGAA	-10.83
26	[a]ccsA(3')-(3')ndhD	118,063-118,105	TTCGAGTTTTTGTTTTGAC**AACCA**GTCAAAACAAAAACTCGAA	-20.22
27	[a]ndhE(5')-(3')ndhG	121,287-121,321	CTTAGAATTTA**TAAATCTTAGAAT**TAAATTCTAAG	-5.7
28	ycf1	128,419-128,464	CATAAATATATGA**GTCCTTCCTATTCCCATAAT**TCATATATTTATG	-6.86
29	ycf1	130,750-130,799	CAATTTTTCTTTG**TTCTGCCAATAAATAACAGTTTTT**CAAAGAAAAATTG	-8.99

[a] Small inversions reported in other studies.

[b] The small inversions and mismatched nucleotides in stem are marked with underlined bold capitals and bold lowercases, respectively.
