# Supplementary material for: The Complete Chloroplast Genome Sequence of Date Palm (Phoenix dactylifera L.)
Source: PLoS One. 2010 Sep 15;5(9):e12762. doi: 10.1371/journal.pone.0012762 (PMC2939885; doi:10.1371/journal.pone.0012762)
Supplement: Table S3 — The intravarietal SNPs found in non-coding regions (0.01 MB PDF) [file pone.0012762.s003.pdf]

Table S3. The intravarietal SNPs found in non-coding regions.

NO.	Gene name	Major(minor) SNP type	Major(minor) Reads number	Major(minor) reads percentage	Position in genome
1	rpoC1[I]	A(C)	249(44)	85.0(15.0)	23477
2	rpoC1[I]	G(T)	846(140)	85.8(14.2)	23725
3	rpoC1[I]	G(C)	659(100)	86.8(13.2)	23765
4	rpoC1[I]	T(G)	551(179)	75.5(24.5)	23967
5	ycf3[I]	T(G)	856(145)	85.5(14.5)	44252
6	ycf3[I]	A(C)	869(99)	89.8(10.2)	44838
7	IGS	C(T)	660(80)	89.2(10.8)	45032
8	IGS	T(G)	500(105)	82.6(17.4)	49604
9	IGS	T(A)	700(80)	89.7(10.3)	65840
10	IGS	A(C)	735(103)	87.7(12.3)	71416
11	IGS	T(G)	574(76)	88.3(11.7)	76129
12	IGS	T(G)	581(76)	88.4(11.6)	76880
13	petB[I]	A(C)	111(32)	77.6(22.4)	77471
14	IGS	A(C)	734(286)	72.0(28.0)	78512
15	IGS	A(C)	525(73)	87.8(12.2)	81676
16	IGS	T(G)	692(93)	88.2(11.8)	122758

IGS, intergenic region; [I] intron region
