# Supplementary figures and images for: The Complete Chloroplast Genome Sequence of Date Palm (Phoenix dactylifera L.)
Source: PLoS One. 2010 Sep 15;5(9):e12762. doi: 10.1371/journal.pone.0012762 (PMC2939885; doi:10.1371/journal.pone.0012762)

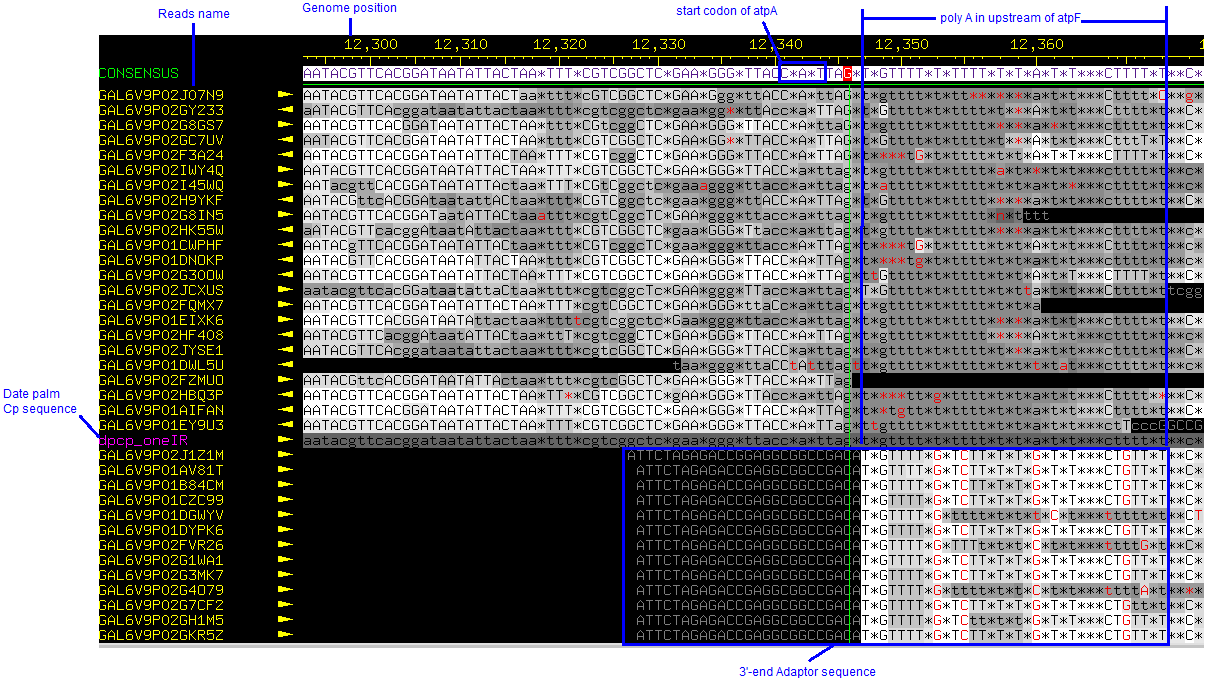

Supplement: Figure S2 — Partial 454 transcription reads alignments with date palm cp genome at downstream of atpF and upstream of atpA. Comments are marked with thick blue line and blue color fonts. (0.25 MB TIF) [file pone.0012762.s005.tif]
